# Uridine supplementation exerts anti-inflammatory and anti-fibrotic effects in an animal model of pulmonary fibrosis

**DOI:** 10.1186/s12931-015-0264-9

**Published:** 2015-09-15

**Authors:** Sanja Cicko, Melanie Grimm, Korcan Ayata, Jessica Beckert, Anja Meyer, Madelon Hossfeld, Gernot Zissel, Marco Idzko, Tobias Müller

**Affiliations:** Department of Pneumology, University Hospital Freiburg, Killianstrasse 6, 79106 Freiburg, Germany; Department of Internal Medicine I, University Hospital RWTH Aachen, Aachen, Germany

## Abstract

**Rationale:**

Pulmonary fibrosis is a progressive disease with only few treatment options available at the moment. Recently, the nucleoside uridine has been shown to exert anti-inflammatory effects in different animal models, e.g. in acute lung injury or bronchial asthma.

**Method:**

Therefore, we investigated the influence of uridine supplementation on inflammation and fibrosis in the classical bleomycin model. Male C57BL/6 mice received an intratracheal injection of bleomycin on day 0 and were treated intraperitoneally with uridine or vehicle. The degree of inflammation and fibrosis was assessed at different time points.

**Results:**

Uridine administration resulted in attenuated inflammation, as demonstrated by reduced leukocytes and pro-inflammatory cytokines in the broncho-alveolar lavage (BAL) fluid. Furthermore, collagen deposition in the lung interstitium was also reduced by uridine supplementation. Similar results were obtained in a model in which animals received repeated intraperitoneal bleomycin injections. In addition uridine inhibited collagen and TGF-ß synthesis by primary lung fibroblasts, the release of pro-inflammatory cytokines by human lung epithelial cells, as well as the production of reactive oxygen species by human neutrophils.

**Conclusion:**

In summary, we were able to show that uridine has potent anti-inflammatory and anti-fibrotic properties. As uridine supplementation has been shown to be well tolerated and safe in humans, this might be a new therapeutic approach for the treatment of fibrotic lung diseases.

## Background

Idiopathic pulmonary fibrosis (IPF) is a disease of unknown origin characterized by progressive loss in lung function leading to respiratory failure with currently no effective treatment options available and therefore a poor prognosis. The pathophysiology of IPF is not fully understood yet. However, injury of alveolar epithelial cells (AECs) paralleled by oxidative stress leading to abnormal activation of fibroblasts is considered to be crucial in this context. The formation of fibroblast and myofibroblast foci via the proliferation of lung fibroblasts, the recruitment of circulating fibrocytes, and epithelial to mesenchymal transition hereby depends on mediators secreted by activated AECs. In summary, these processes result in excessive deposition of collagen, fibronectin and other components of the extracellular matrix in the lung parenchyma [1–3].

Besides their well-characterized role in cell metabolism, the involvement of different nucleosides in particular adenosine in different inflammatory disorders has been demonstrated extensively. Adenosine-induced overproduction of IL-13 results in pulmonary fibrosis, respiratory failure and death in a transgenic mouse model [4]. Inosine in contrast has been found to be protective in animal models of acute lung injury or bronchial asthma in an adenosine receptor-dependent manner [5, 6]. In contrast no receptor has been identified for the nucleoside uridin so far which has also been demonstrated to have anti-inflammatory properties [7]. Recently we were able to show that the exogenous application of uridine results in decreased allergic airway inflammation in both OVA- and house dust mite induced asthma. Though the mechanisms behind these findings could not be fully resolved an effect on lung epithelial cells could be shown [8]. Additionally, uridine is a potent inhibitor of leucocyte adhesion [9]. Interestingly, oral uridine supplementation is a safe, well tolerated and efficacious treatment to reduce mitochondrial toxicity caused by highly active antiretroviral therapy (HAART) in humans [10, 11].

In this study we investigated therapeutic properties of uridine in the classical animal model of bleomycin-induced lung injury and fibrosis.

## Material and methods

### Animals

C57Bl/6 mice, were bred at the animal facility at the University of Freiburg. All experiments were performed according to institutional guidelines of the local animal ethics committee (Regierungspräsidium Freiburg).

### Bleomycin model of pulmonary fibrosis (intratracheal model)

Male C57BL/6 animals were anaesthetized by i.p. ketamine/xylazine administration and received an intratracheal (i.t.) injection of bleomycin (80 μl, 1 mg/ml) or vehicle (saline) as a negative control. Animals were treated with uridine (80 μl, 24 μg/ml; Sigma Aldrich, Germany) or vehicle at the indicated time points. Mice were sacrificed at different time points (see results section) via i. p. injection of thiopental. BAL was performed with 3 × 1ml of Ca^2+^ and Mg^2+^ free PBS (Gibco, Paisley, UK) supplemented with 0.1 mM sodium EDTA (Sigma Aldrich, Germany), followed by lung resection and storage in OCT freezing medium. BAL cells were counted, differential cell counts were done by FACS analysis, as described previously [12]. Frozen lung sections were stained with hematoxylin and eosin for histological analysis.

### Bleomycin model of pulmonary fibrosis (intraperitoneal model)

Male C57BL/6 animals received i.p. injections of bleomycin (140 μl, 6 mg/ml) or vehicle (saline) as a negative control twice a week over 4 weeks. Starting from day 14 on animals were treated intraperitoneally with uridine (200 μl, 2,4 mg/ml) or vehicle 3 times a week. On day 30 animals were killed and BAL was performed and analysed as described above. Frozen lung sections were stained with hematoxylin and eosin for histological analysis.

### Mediator measurements in BALF

BALF cytokine contents were determined by ELISA (R&D Systems, Minneapolis, USA), as described by the manufacturer. BALF collagen content was measured by Sircol assay (Biocolor, Carrickfergus, UK).

### Collagen quantification in histological lung slides

Frozen lung sections were incubated in picrosirius red solution (0,2 gr of Picosirius Red diluted in 100 ml of 1,2 % picric acid, both Sigma-Aldrich), for one hour. After washing with water, tissue sections were stained with hematoxylin for 5–10 s. Slides were washed with running tap water and dehydrated in 70 %, 90 % and absolute ethanol, followed by xylene. Entellan (Merck) was used to mount the coverslip. Images were obtained using Axio Lab.A1 microscope (Zeiss) with 200× magnification and AxioCam ICc1 (Zeiss). Collagen quantification was made with ImageJ.

### Cell culture

A549 cells were grown in RPMI1640-Medium (Gibco, Paisley, UK) containing 10 % fetal calf serum (FCS; Biochrom, Berlin, Germany) and 1 % penicillin/streptomycin (P/S; Gibco, Paisley, UK) in cell culture flasks (BD Falcon, Bedford, MA) at 37 °C, 5 % CO2, and 100 % humidity. For subculture cells were trypsinized and seeded into 24-well tissue culture plates at a density of 2 × 10^5^ cells/well. After 2 h, medium was changed and cells were stimulated as indicated. After additional 8 h, cell supernatants were collected and analyzed by ELISA.

For the isolation of primary lung fibroblasts lungs of C57Bl/6 mice were excised and cut in small pieces, followed by digestion with collagenase. Single cell suspensions were obtained by passing through a cell strainer. Cells were grown in RPMI1640-Medium (Gibco, Paisley, UK) (supplemented 10 % FCS and 1 % P/S) in cell culture flasks (BD Falcon, Bedford, MA) at 37 °C, 5 % CO_2_ in a humidified atmosphere. Medium was changed every 2 or 3 days. Cells were trypsinized after 14 days and used for experiments. Purify of isolated cells was assessed by microscopy. The cells were seeded into 6-well tissue culture plates at a density of 0,5 × 10^6^ cells/well. After 24h, medium was changed and cells were stimulated with Bleomycin (1 μg/ml) ± uridine. After 24 h, total RNA of these cells was isolated with Qiazol (QIAGEN GmbH, Hilden, Germany) according to the manufacturer’s protocol.

### Isolation of human neutrophils and measurement of reactive oxygen species (ROS)

Neutrophils from healthy volunteers were isolated from venous blood using a Pancoll gradient (PAN-Biotech GmbH, Aidenbach, Germany) as described previously [13]. Resulting neutrophils were resuspended in PBS. Neutrophils were seeded in 96-well plates (1 × 10^5^/well), in RPMI1640 medium supplemented with 10 % FCS and penicillin/streptomycin, and incubated at 37 °C, 5 % CO2 in a humidified atmosphere. Neutrophils were preincubated with uridine (10^−5^ mol/l) or vehicle for 15 min, then 50 μl of lucigenin (2 mg/ml) was added. Finally, 10 μl of Bleomycin (1 μg/ml) were added to the cells and the stimulated generation of ROS was measured by a computerized chemoluminometer at 37 °C (Berthold Technologies LP96V, Germany).

### Quantitative PCR

Quantitative PCR was performed on LightCyler 480 (Roche) using the fast-blue + UNG kit (Eurogentec). Collagen, TGF-β and ß2-microglobulin primers/probes were designed using Beacon Designer v 7.50 (Premier Biosoft). Percent reference gene (RG) values for the gene of interest (GOI) were calculated using the formula: % RG = 100 × 2(−ΔCt). Cumulative standard deviations were calculated using the formula: SD = 100 × 2(−ΔCt) × ((ln2 × SDRG)2 + (ln2 × SDGOI)2)1/2 [12].

### Statistical analysis

If not stated otherwise, groups were compared using anova, followed by Bonferroni comparison test. Probability values of *p* < 0.05 were regarded as significant.

## Results

### Uridine supplementation decreases inflammation in the early phase after intratracheal bleomycin administration

**Fig. 1:**
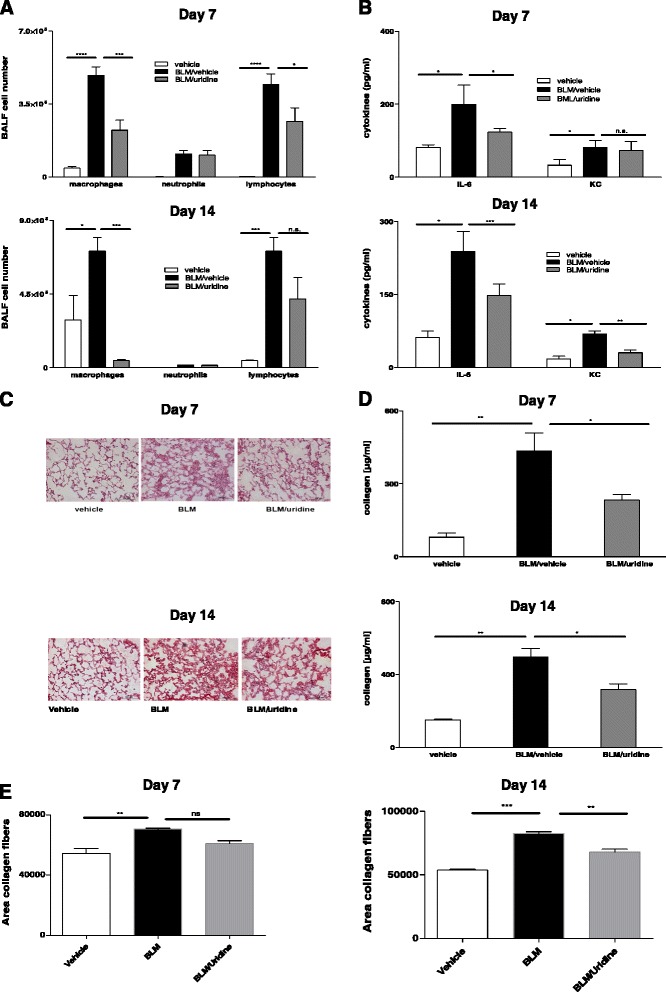
Uridine supplementation decreases inflammation in the early phase after intratracheal bleomycin administration. Animals were treated with i. t. bleomycin on day 0. Uridine or vehicle was administered after 6 h, at day 5 and at day 10. Animals were assessed for inflammation and fibrosis at day 7 and day 14. **a** BALF differential cell counts. **b** BALF cytokine levels. **c** HE stainings of lung sections. **d** BALF collagen content. **e** Collagen content on histological lung slides. Data are means ± SEM, *n* = 4–6 mice per group. *p < 0.05; **p < 0.01; ***p < 0.001; ****p < 0,0001. The experiment was repeated twice with similar results

To investigate the influence of uridine supplementation on the course of fibrotic lung disease we used the well-established animal model of bleomycin induced lung injury/fibrosis. In the prophylactic protocol mice received an intratracheal injection of bleomycin or vehicle on day 0. Animals were treated intraperitoneally with uridine or vehicle after 6 h, on day 5 and on day 10 following bleomycin administration. Animals were sacrificed on day 7 and day 14 and assessed for inflammation and fibrosis. Treatment with uridine resulted in decreased inflammation, as shown by reduced inflammatory cells in the broncho alveolar lavage (BAL) fluid (Fig. 1a). This was paralleled by decreased BAL fluid cytokine levels and by attenuated inflammation on histological lung sections (Fig. 1b, c). In addition, the collagen content in the BAL fluid was lower in the animals treated with uridine (Fig. 1d).

### Uridine supplementation decreases inflammation and fibrosis in the late phase after intratracheal bleomycin administration

**Fig. 2:**
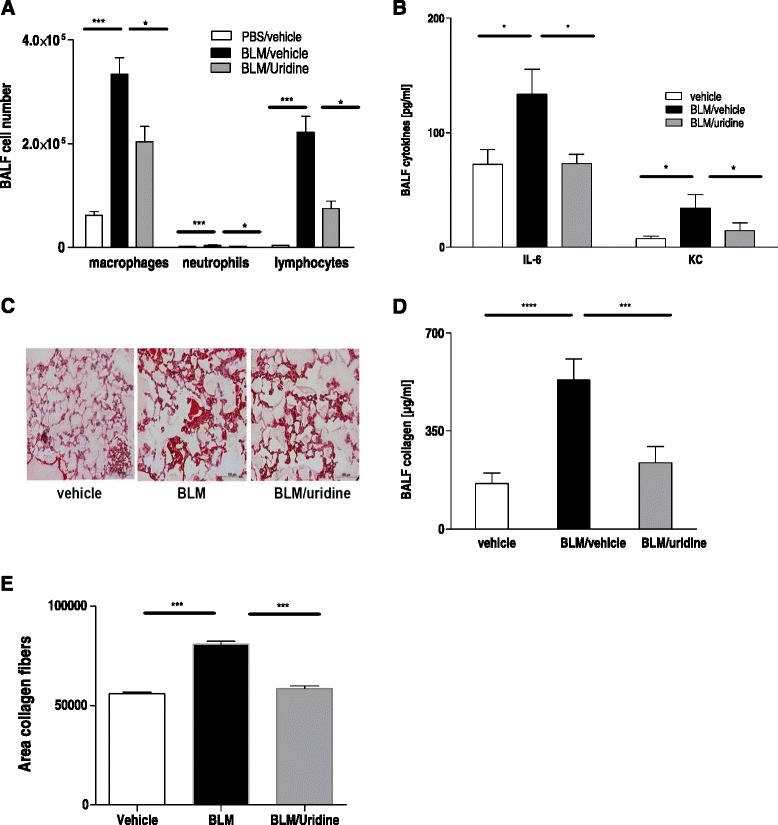
Uridine supplementation decreases inflammation and fibrosis in the late phase after intratracheal bleomycin administration. Animals were treated with i. t. bleomycin on day 0. Uridine or vehicle was administered three times a week starting from day 14. Animals were assessed for inflammation and fibrosis at day 30. **a** BALF differential cell counts. **b** BALF cytokine levels. **c** HE stainings of lung sections. **d** BALF collagen content. **e** Collagen content on histological lung slides. Data are means ± SEM, *n* = 4–6 mice per group. *p < 0.05; **p < 0.01; ***p < 0.001; ****p <  0,0001. The experiment was repeated twice with similar results

As uridine was able to decrease inflammation and fibrosis when administered early after bleomycin we next questioned whether uridine administration is also protective during the fibrotic phase. Therefore, bleomycin or vehicle was injected intratracheally on day 0. Starting from day 14 mice were treated 3 times a week with either uridine or vehicle. The degree of inflammation and fibrosis was determined on day 30. As shown in Fig. 2a, uridine treatment significantly reduced total BAL cell count and the number of macrophages and lymphocytes. Additionally, the concentration of pro-inflammatory and pro-fibrotic cytokines was lower in the BAL fluid derived from uridine-treated animals (Fig. 2b). Furthermore, we observed reduced collagen deposition in the airspaces and decreased collagen levels in the BAL fluid (Fig. 2c, d).

### Uridine supplementation inhibits inflammation and fibrosis following chronic bleomycin administration

**Fig. 3:**
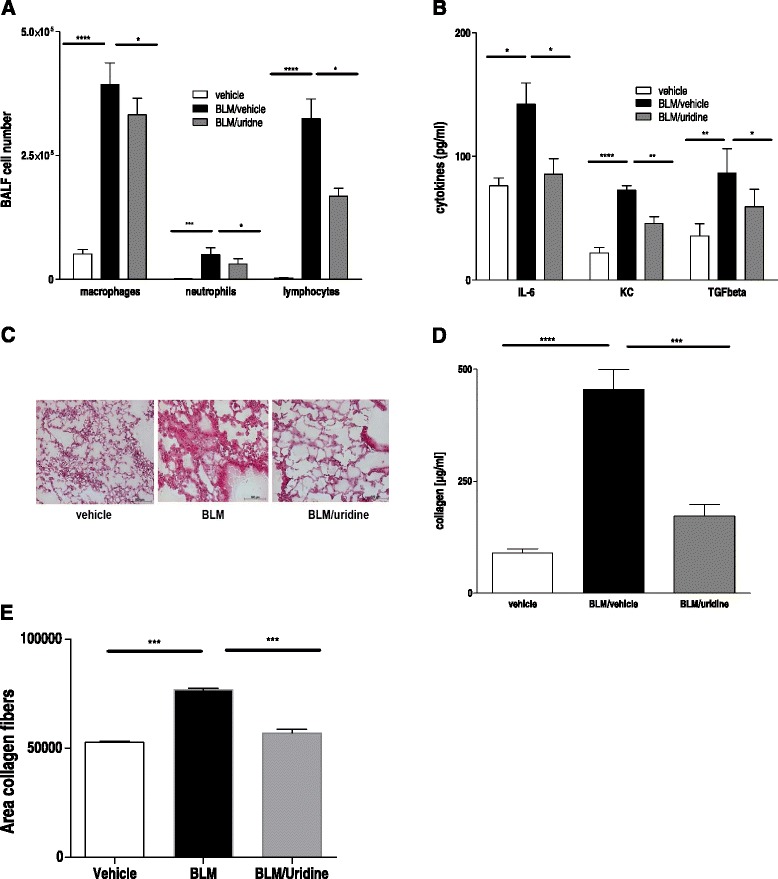
Uridine supplementation inhibits inflammation and fibrosis following chronic bleomycin administration. Animals were treated with i. p. bleomycin twice a week over a time period of 4 weeks. Starting from day 14 on animals received either uridine or vehicle for 3 times a week. Animals were assessed for inflammation and fibrosis at day 30. **a** BALF differential cell counts. **b** BALF cytokine levels. **c** HE stainings of lung sections. **d** BALF collagen content. **e** Collagen content on histological lung slides. Data are means ± SEM, *n* = 4–9 mice per group. *p < 0.05; **p < 0.01; ***p < 0.001; ****p < 0,0001. The experiment was repeated once with similar results

In contrast to IPF in humans, pulmonary fibrosis after a single intratracheal bleomycin administration is known to be self-limiting. Hence, another model in which mice were repeatedly exposed to intraperitoneal bleomycin was used. This model is characterized by long lasting fibrosis and subpleural scaring, a pattern similar to usual interstitial pneumonia (UIP) in humans [14]. Therefore, animals received intraperitoneal bleomycin injections twice a week over 4 weeks. Treatment with uridine which was started at day 15 was associated with a strong reduction in inflammation as demonstrated by reduced inflammatory cells and pro-inflammatory cytokines in the BAL fluid (Fig. 3a, b). Moreover, histological analysis showed decreased tissue fibrosis paralleled by lower collagen contents in the BAL fluid of uridine treated animals (Fig. 3c, d).

### Uridine inhibits the release of pro-inflammatory mediators by human lung epithelial cells

**Fig. 4:**
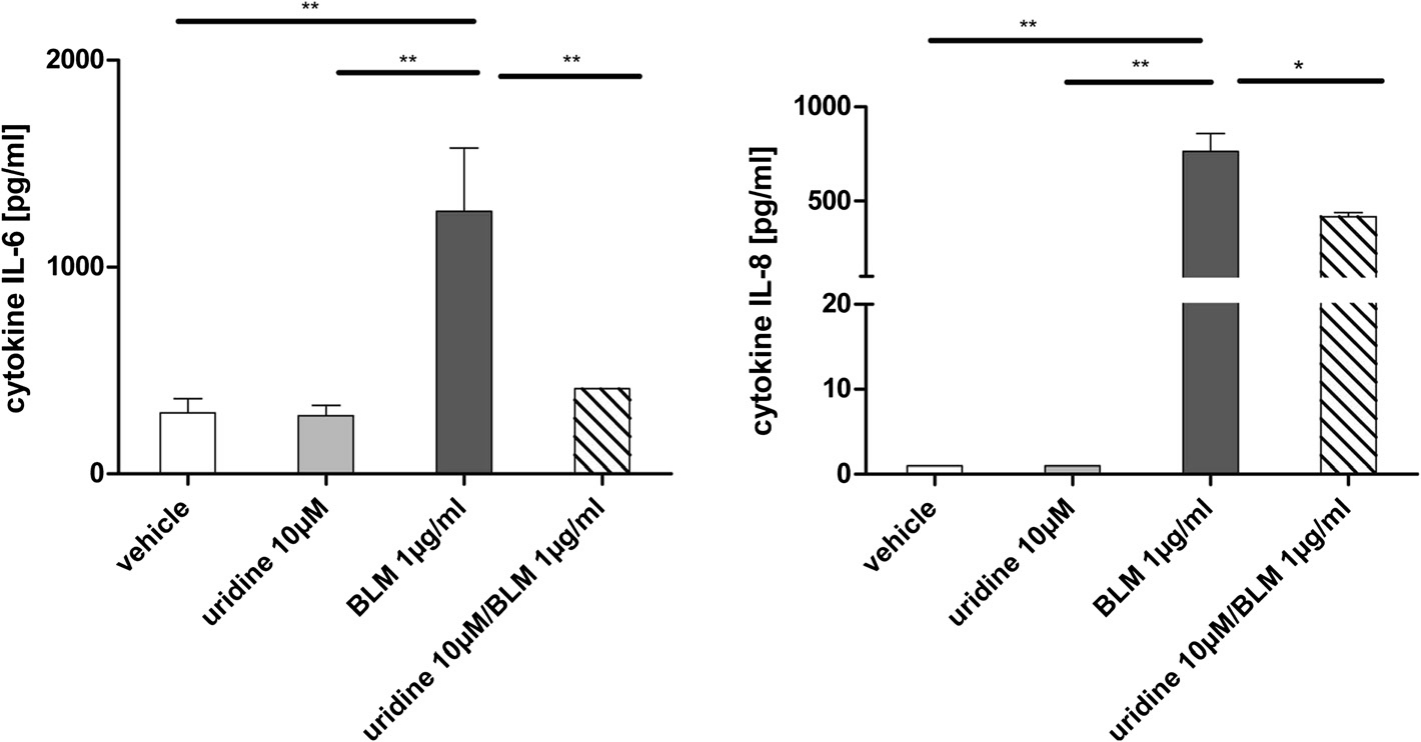
Uridine inhibits the release of pro-inflammatory mediators by human lung epithelial cells. A549 cells were stimulated with bleomycin (1 μg/ml) ± uridine. After an incubation period of 24 h levels of IL-6 and IL-8 in cell culture supernatants were determined by ELISA. Data are means ± SEM, *n* = 3. *p < 0.05; **p < 0.01

Alveolar epithelial cells are known to play a pivotal role in the pathogenesis of fibrotic lung diseases [1]. In pursuit of the mechanisms behind our findings we investigated the effects of uridine on the production of the pro-inflammatory cytokines IL-6 and IL-8 by A549 cells, a cell line resembling human alveolar epithelial cells type 2. As shown in Fig. 4, uridine was able to suppress the bleomycin induced release of both IL-6 and IL-8 in a dose dependent manner.

### Uridine inhibits collagen and TGF-β production by primary lung fibroblasts

**Fig. 5:**
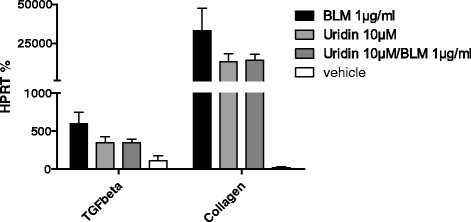
Uridine inhibits collagen and TGF-β production by primary lung fibroblasts. Fibroblasts were stimulated as indicated for 8 h, total RNA was isolated. The mRNA specific for collagen and TGF-β was quantified as described. Data are means ± SEM, *n* = 3. *p < 0.05; **p < 0.01; ***p < 0.001

Excessive production and deposition of extracellular matrix components by lung fibroblasts is crucial for the development of pulmonary fibrosis. In addition fibroblasts interact with inflammatory and lung structural cells and are capable of releasing different cytokines and growth factors [2]. As demonstrated in Fig. 5, uridine decreased collagen and TGF-ß mRNA synthesis by primary lung fibroblasts.

### Uridine inhibits production of reactive oxygen species by human neutrophils

**Fig. 6:**
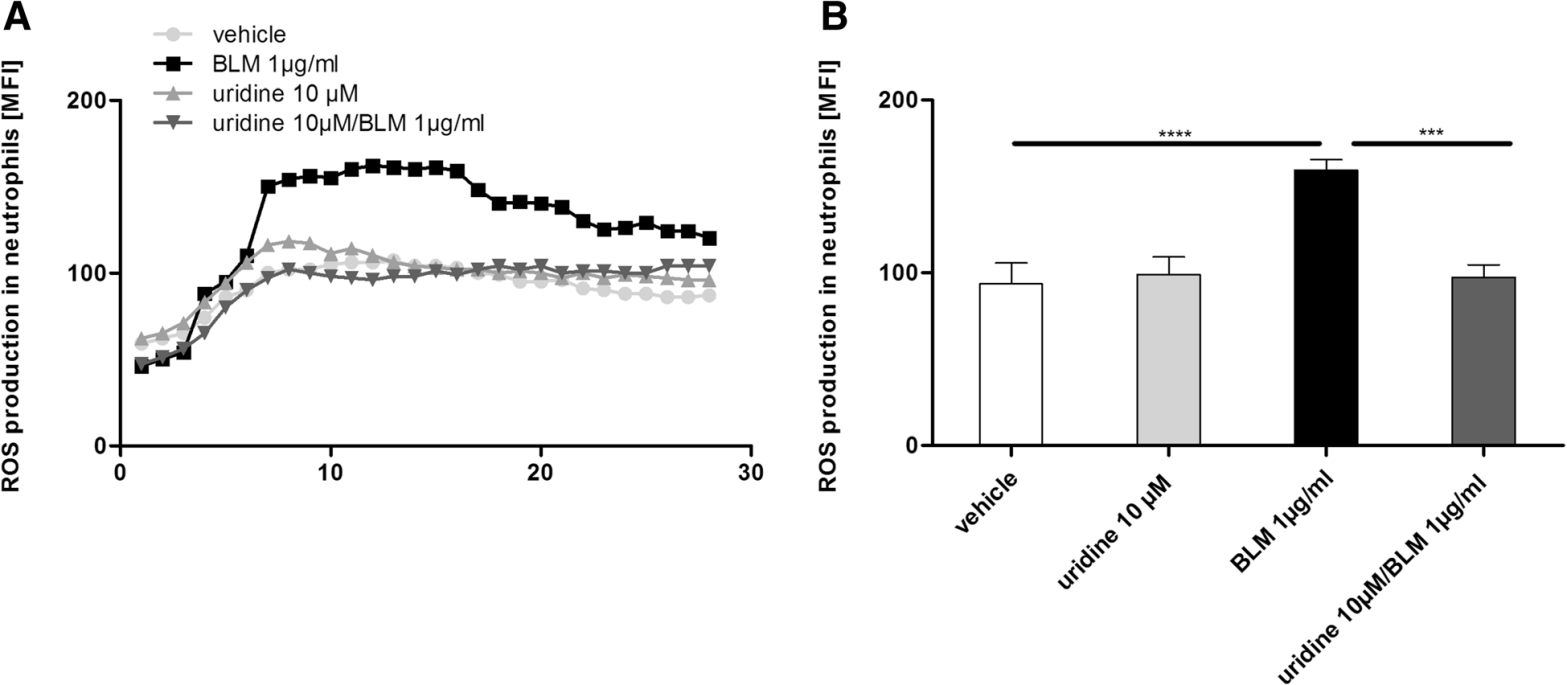
Uridine inhibits production of reactive oxygen species by human neutrophils. Neutrophils were stimulated as indicated and lucigenin-dependent chemiluminescence was measured. **a** Time courses upon stimulation with vehicle, bleomycin, and bleomycin + uridine. One representative out of 8 experiments is shown. **b** Maximal chemoluminescence after stimulation with vehicle, bleomycin, and bleomycin + uridine. Data are means ± SEM, *n* = 7. *p < 0.05; **p < 0.01; ***p < 0.001; ****p< 0,0001

The importance of oxidative stress in the pathophysiology of pulmonary fibrosis has been demonstrated extensively [2]. Thus we sought to determine whether uridine can influence the production of reactive oxygen species by human neutrophils. As shown in Fig. 6, exposure to bleomycin resulted in transient ROS production. However, pre-treatment with uridine significantly inhibited ROS production by neutrophils.

## Discussion

The involvement of the nucleoside adenosine in the pathogenesis of inflammatory lung disorders, including pulmonary fibrosis has been studied extensively [4, 15, 16]. Recently, we were able to demonstrate that local administration of uridine inhibited the cardinal features of bronchial asthma [8]. In this study we investigated whether uridine supplementation can also influence the course of fibrotic lung disease.

In an acute model of bleomycin induced lung injury intraperitoneal treatment with uridine resulted in decreased inflammation and collagen deposition. These results are in line with previous studies showing that uridine administration is protective in Sephadex- or allergen-induced lung inflammation [7, 8, 17]. However, beyond dampening inflammation following acute bleomycin administration uridine was also able to suppress tissue fibrosis at later time points. Furthermore, fibrotic changes were also reduced by uridine when bleomycin was administered repeatedly. This animal model is characterized by longer lasting fibrosis with subpleural predominance. Hence it is supposed to be closer to idiopathic pulmonary fibrosis in humans than the acute model [14]. To our best knowledge this is the first study showing that uridine has both anti-inflammatory and anti-fibrotic properties.

A receptor specific for uridine has not been identified, yet. Consequently, it has been hypothesized that uridine might exert its effects indirectly via the generation of UDP and UTP being able to bind to the purinergic receptors P2Y_2_, P2Y_4_, and P2Y_6_. However, both P2Y_2_ and P2Y_6_ receptor subtypes have actually been demonstrated to mediate pro-inflammatory effects in bronchial asthma or chronic obstructive pulmonary disease [12, 18, 19]. Another possibility might be the interaction of uridine with adenosine receptors. In accordance with this a previous study demonstrated that uridine can actually activate A_1_ receptors [20]. Though there is evidence that A_1_ receptors exhibit both pro- and anti-inflammatory activities, most studies suggest that the activation of A_1_ receptors is in summary pro-inflammatory [21, 22]. Moreover, deficiency in adenosine deaminase resulting in increased pulmonary adenosine levels actually leads to a fibrotic lung disease. Additionally, in a previous study we were able to demonstrate that the anti-inflammatory effects of uridine on lung epithelial cells were not dependent on adenosine receptors [8]. Therefore, taken together the anti-inflammatory and anti-fibrotic effects of uridine are most likely independent of adenosine receptors.

The subsequent release of pro-inflammatory and pro-fibrotic cytokines/chemokines after epithelial cell injury is a crucial step in the pathogenesis of fibrotic lung diseases [1]. In this study we were able to demonstrate that when co-administered with bleomycin uridin inhibited the release of the pro-inflammatory cytokines IL-6 and IL-8 by human alveolar epithelial cells. The latter one is considered as the key chemokine for the recruitment and activation of neutrophils [23]. Interestingly, an association between a high BAL fluid neutrophil count and a poor prognosis in IPF has been found [24]. Apart from inflammation IL-8 has been identified as a pro-angiogenic factor in the context of idiopathic pulmonary fibrosis [25]. IL-6 is a pleiotropic cytokine whose importance in various lung disorders including pulmonary fibrosis has been demonstrated extensively [15, 26]. Hence, uridine might influence the course of fibrotic lung disease by altered cytokine secretion of epithelial cells. However, in vivo altered cytokine secretion after uridine administration might not be limited exclusively to airway epithelial cells and indirect effects of uridine might also be of importance. In a line with these findings, uridine was also able to decrease TGF-ß and collagen synthesis by primary lung fibroblasts. The production and deposition of collagen and other extracellular matrix components is a characteristic feature of pulmonary fibrosis. In this context TGF-ß has been identified as an important growth factor, mediating e.g. epithelial to mesenchymal transition [27].

Though the exact role of oxygen radicals in the pathophysiology of IPF remains controversial increased markers of oxidative stress have been found in IPF patients [28]. Thus decreased production of reactive oxygen species might contribute to reduced pulmonary inflammation and fibrosis in uridine treated animals.

As mentioned earlier in this manuscript treatment with uridine has been shown to abrogate mitochondrial toxicity induced by anti-retroviral therapy in patients infected with HIV [10, 11]. Recently, mitochondrial dysfunction has gained attention as a mechanism contributing to epithelial cell injury and the progression of pulmonary fibrosis [29, 30]. Hence, uridine supplementation might lead to an improved mitochondrial function thereby limiting pulmonary inflammation and fibrosis.

Nucleosides are rapidly metabolized in the extracellular space. Adenosine, e.g. is characterized by an extremely short half-life when administered intravenously [31]. Hence the pharmacokinetic characteristics of uridine have to be taken in account in this context. However, previous studies were able to demonstrate that the half-life of uridine in plasma is considerably higher compared to adenosine [32, 33]. Accordingly, uridine plasma levels after intraperitoneal administration appear to be high enough to exert anti-inflammatory and anti-fibrotic effects.

In summary we demonstrated that uridine inhibits inflammation and fibrosis in bleomycin induced lung injury. As a receptor specific for uridine has not been identified yet, the exact mechanisms behind these findings are not completely understood at the moment. Nevertheless, uridine inhibited the production of collagen and pro-inflamamtory/fibrotic cytokines by alveolar epithelial cells and lung fibroblasts, as well as the release of reactive oxygen species by activated neutrophils. Interestingly, uridine supplementation has been found to be safe and well tolerated in humans [10, 11]. Therefore, further studies investigating the impact of uridine administration on the course of fibrotic lung diseases in humans would be of great interest.

## Conclusions

Uridine which can be safely administered in humans is a potent inhibitor of bleomycin-induced pulmonary inflammation and fibrosis. Consecutively, though further research is still needed, this might be a new approach for the treatment of fibrotic lung diseases in humans.
